# Noise Exposure and Self-reported Hearing Impairment among Gas-fired Electric Plant Workers in Tanzania

**DOI:** 10.29024/aogh.2305

**Published:** 2018-10-09

**Authors:** Witness John, Gloria Sakwari, Simon Hendry Mamuya

**Affiliations:** 1 Department of Environmental and Occupational Health, Muhimbili University of Health and Allied Sciences, TZ

## Abstract

**Background:**

Gas-fired electric plants are equipped with heavy machines, which produce hazards including noise pollution. Exposure to high level of noise of above 85dB(A) is known to bring about Noise-Induced Hearing Loss (NIHL). This study aimed to assess noise exposure level and reported prevalence of noise-induced hearing loss among workers in gas-fired electric plants.

**Material and Methods:**

This cross-sectional study was conducted in three gas-fired electric plants in Dar es Salaam (Plant A, Plant B and Plant C) from July to August 2017. A noise logging dosimeter was used to measure personal noise exposure level. A questionnaire was used to collect information on managerial factors, individual factors, socio-demographic factors and history of the participants. A short screening validated questionnaire was used to obtain noise exposure score. Frequency distribution, Chi-square test and Regression analyses were done using SPSS version 20.

**Results:**

One hundred and six participants were involved in the study. Noise exposure level among gas-fired electric plant workers was above 85dB(A), n = 37. The equivalent sound level (LAeq) measured over 8 hours was (98.6 ± 9.7) dB(A). The mean noise peak level was (139.5 ± 9.4) dB(A). Plant C had higher mean noise exposure level (TWA) of (96.9 ± 5.1) dB(A) compared to plant B 96.4 ± 3.7dB(A) and plant A 78.7 ± 11.9dB(A). Participants in both operation and maintenance had higher equivalent sound level (LAeq) measured over eight hours of 101.980 ± 3.6dB(A) compared to maintenance alone 98.5 ± 12.4dB (A) or operation 97.7 ± 8.8dB (A). Proportion of participants with reported hearing loss was 57(53.8%) where 44(41.5%) participants reported difficulty hearing people during conversations. Hearing protective devices (HPDs) were reported to be used by a majority, 101(95.3%).

**Conclusion:**

Workers in gas-fired plants are exposed to high noise levels that could damage their hearing. Hearing conservation programs should be established and maintained in this work environment.

## Introduction

Noise pollution has been recognized as one of the major threats to human well-being. It has been shown that noise, in extreme ranges, can damage hearing and can be classified as a hazard [1]. Noise, in addition to causing hearing loss, has also been implicated in detrimental impact on human physiological and psychological systems including a multitude of bodily stress responses [2]. Noise exposure in the occupational setting is increasing. The liability and rising costs associated with occupational noise has increased pressure on numerous industries to reduce and alleviate this problem.

The effect of exposure to noise in relation to the intensity as well as frequency characteristics of the noise was investigated in two Tanzanian textile mills. A peak noise level above the threshold limit value 85dB(A) at a hazardous frequency range of 2500–5000 Hz was recorded [3]. Another study addressed the noise levels and factors that influence noise pollution in two small-scale wood and metal industries in Tanzania [4]. The results show that both sites exhibited equivalent noise levels higher than 90dB(A), exceeding the permissible occupational exposure level limit.

Noise induced hearing loss (NIHL) generally occurs over a long period of exposure to high noise levels, developing unnoticeably and gradually as time passes [5]. It has been shown that the detrimental effects from noise are 100% preventable [6]. Noise exposures contributing to NIHL can be continuous or intermittent in nature and cause hearing loss as a result of damage to the hair-like cells structure of the cochlea [7]. NIHL is prevalent across industries and countries [8], as hearing loss accounts for approximately one third of the occupational illnesses reported in Europe [9]. In a study on occupational noise exposure, 23% of workers who were exposed to noise presented with difficulty in hearing, compared with only 7% of those not exposed [10].

A study of electrical workers, often viewed as the least exposed of the trades, resulted in exposures over the OSHA’s Allowable Limit (AL) of 85dB(A) in 25% of dosimeter samples and exceeded the PEL of 90dB(A) in 5% of samples [11].

High prevalence of hearing loss among workers exposed to hazardous noise have been reported in various industries worldwide [12]. It is estimated that the global burden of hearing impairment due to occupational noise exposure is16% [13]. In sub-region AFR E, according to the WHO sub-region classification where Tanzania is included, the burden of hearing impairment attributed by occupation is estimated to be 18%, where 23% is for male and 12% for female workers [13]. Workers in gas-fired electric plants are exposed to high noise over a working period at different levels depending on the task and work station.

Another cross-sectional study done in the steel industry in Iran showed that 41.3 percent of employees had standard threshold shift in both ears, and there was a significant relationship between the noise exposure level and work experience with standard threshold shift [14].

There is significant association between hearing loss and cardiovascular disease, diabetes, high blood pressure, smoking, lack of exercise and high cholesterol [15]. Diabetes and age were also reported to predict hearing loss. Diabetics had a greater hearing loss than non-diabetics in the 3–6 kHz range in a major US population survey [16]. Other studies have shown illnesses at childhood may predispose a person to hearing loss [17].

Most occupational diseases are preventable by different methods such as engineering controls, administrative measures and personal protective equipment(PPEs) [18]. However, adherence to use of PPE has been shown to be low in most occupational exposures. There are different reasons for workers not wearing HPDs; that is, lack of knowledge, concern that it may impair ability to communicate, discomfort, and lack of availability [19,20]. However, hearing protection devices are a last-resort measure; they are used when other control measures are insufficient.

In Tanzania, noise exposure and the reported prevalence of noise-induced hearing loss among workers in gas-fired electric plants was not known. Therefore, this study aimed to determine employee noise exposure level and reported prevalence of noise-induced hearing loss among workers in gas-fired electric plants so as to put in place proper control strategies. This study may provide information that can be used by other researchers in the future in solving noise exposure problems in the working environment.

## Methodology

### Study Area And Population

This was a cross-sectional conducted in three gas-fired electric plants (named A, B and C) in Dar es Salaam. Plant A has an installed capacity of 105 Megawatts (MW) consisting of three open cycle gas turbines, SGT–800 industrial type turbines of 35 MW each. Plant B is installed with five generating units giving a total installed capacity of 45 MW. Plant C is installed with twelve generating engines each with a capacity of 8.73 MW totaling an installed capacity of 104 MW. All plants use natural gas in their operations.

Participants were randomly selected from administrative list in each plant based on the number of workers in operation and maintenance departments. Proportional sampling method was used to insure weighted participation from each plant. Using OpenEpi software and a proportional of 16% prevalence of noise-induced hearing loss reported by Ivera and colleagues [25] the total number of participants needed was 106.

### Data Collection Techniques

A questionnaire was used to collect data on socio-demographic factors, managerial factors and individual factors such as use of hearing protection devices, behavior on hearing protection devices, and feeling of hearing loss. A short screening validated questionnaire was used to obtain a noise exposure score [21]. The first question states, “How often were you around areas with loud sounds such as club, drilling machines, blasting of rocks and firearms such as rifles, pistols, shotguns?” The second question states, “How often were you exposed to loud sounds while working on a paid job?” The third question states, “How often were you exposed to any other types of loud sounds, such as power tools, lawn equipment, or loud music?” These questions had five responses and a score of 0 was given for those never exposed, the score of 1 was given for those exposed every few months, the score of 2 was given for those exposed monthly, the score of 3 was given for those exposed weekly and the score of 4 was given for those exposed daily. The sum of score for a person answering all questions is 12 hence the scores would range from 0–12. The scores were categorized in low noise exposure for those scoring a total of 0–4 and high noise exposure risk for 5–12 scores.

Noise exposure scores were divided in to two groups.

- Noise exposure score in a range of 0–4 is termed as low noise risk, where a participant’s risk of developing noise-induced hearing loss is relatively low if the participant continues to experience similar levels of noise in the future. However, if noise exposures increase, the risk of developing hearing loss will increase as well.
- Noise exposure score ranging from 5 and above is termed as high noise risk, where a participant is at the risk of developing noise-induced hearing loss if the participant continues to experience similar or higher levels of noise in the future.

Noise logging dosimeters were used to measure noise exposure level (TWA, LAeq and Peak level) among workers in three of the gas-fired electric plants.

Data was analyzed using Statistical Package for Social Sciences SPSS version 20. Univariate analysis, bivariate analysis and multiple logistic regression analysis was done. P-value less than 0.05 was considered to indicate statistically significant association between dependent variables and independent variables.

## Results

A total of 106 workers out of 160 employees in the three gas-fired electric plants participated in this study. Workers in plant A were younger than those in plants B and C. A majority of the participants from the three plants were male (n = 101) (Table 1).

**Table 1:** Socio-demographic characteristics of the study population (n = 106).

Characteristics	Plant A	Plant B	Plant C

Age (AM (SD)) years	36.5(10.0)	42.7(11.7)	43.4(12.2)

**Age group (n (%))**			
20–29	9(28.1)	6(18.2)	8(19.5)
30–39	13(40.6)	8(24.2)	7(17.1)
40–49	5(15.6)	4(12.1)	6(14.6)
50–59	5(15.6)	15(45.5)	20(48.8)

**Sex (n (%))**			
Male	30(93.8)	31(93.9)	40(97.6)
Female	2(6.2)	2(6.1)	1(2.4)

**Marital status (n (%))**			
Single	10(31.2)	7(21.2)	8(19.5)
Married	22(68.8)	25(75.8)	32(78.0)
Widowed	0(0.0)	1(3.0)	1(2.4)

**Educational Level (n (%))**			
Primary	0(0.0)	2(6.1)	7(17.1)
Secondary	1(3.1)	5(15.2)	4(9.8)
Tertiary	31(96.9)	26(78.8)	30(73.2)

### Personal Noise Exposure Level and Noise Exposure Score

**Figure 1:**
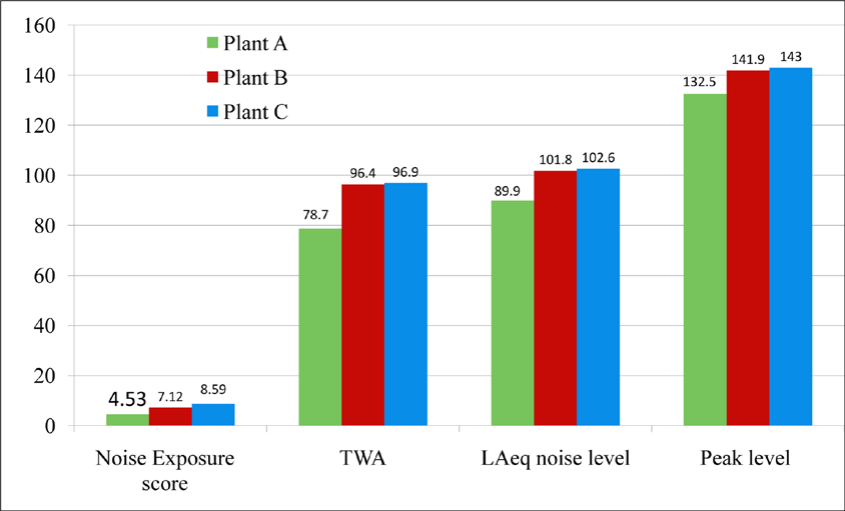
Noise exposure score and Personal noise exposure levels in dB(A).

The arithmetic mean of noise exposure level (TWA) was 91.3dB(A). The mean equivalent sound level (LAeq) measured over eight hours was (98.6 ± 9.7) dB(A). The mean noise peak level was (139.5 ± 9.4) dB(A). The arithmetic mean score for reported noise exposure score for all the gas-fired electric plant is AM(SD) is 6.9(3.3) (Figure 1). There was a significant correlation between noise score and noise exposure level among gas-fired electric plant workers (r = 0.409 and p = 0.012).

### Noise Exposure Level and Noise Exposure Score in Work Section

Participants in both the operation and maintenance departments had a higher mean equivalent sound level (LAeq) of 101.980 ± 3.6dB(A); the maintenance department had a mean equivalent sound level (LAeq) measured over eight hours of 98.5 ± 12.4dB(A), and the operation department had a mean equivalent sound level (LAeq) measured over eight hours of 97.7 ± 8.8dB(A) (Table 2).

**Table 2:** Noise exposure level and noise exposure score in work section among gas-fired electric plant workers.

		n	Mean	Std. Deviation	95% CI for Mean	

					Lower Bound	Upper Bound

Noise exposure level(dBA)	Maintenance	14	89.643	13.95	81.590	97.696
	Operation	18	91.189	9.89	86.268	96.109
	Operation and Maintenance	5	96.660	4.03	91.658	101.662

LAeq noise Level(dBA)	Maintenance	14	98.529	12.40	91.368	105.690
	Operation	18	97.717	8.7939	93.344	102.090
	Operation and Maintenance	5	101.980	3.5647	97.554	106.406

Peak level(dBA)	Maintenance	14	137.050	14.4332	128.717	145.383
	Operation	18	141.172	3.6200	139.372	142.972
	Operation and Maintenance	5	140.660	4.4168	135.176	146.144
	Maintenance	61	6.85	3.478	5.96	7.74

Noise exposure score	Operation	37	7.16	3.149	6.11	8.21
	Operation and Maintenance	8	6.13	2.588	3.96	8.29

### Specific Task and Reported Noise Exposure Score among Gas-Fired Electric Plants

The mean noise score for plant attendants was higher, 8.6 ± 2.3, artisans had a mean score of 8.0 ± 2.7 and engineers had the lowest mean noise score of 5.1 ± 2.9. Artisans had a high mean noise exposure level (TWA) of 98.2 ± 2.3dB(A), whereas the engineers had the lowest noise exposure level (TWA) of 78.5 ± 15.0dB(A). The noise peak level for all the tasks was above 85dBA, which shows that there was no a safe task, even though their noise score and noise exposure level was less than others in comparison (Table 3).

**Table 3:** Specific task, reported noise exposure score, noise exposure level (TWA) and peak noise level among gas-fired electric plant.

		n	Mean	Std. Deviation	95% CI for Mean	

					Lower Bound	Upper Bound

Noise score	Shift Supervisor	13	7.31	3.637	5.11	9.51
	Technician	45	6.36	3.439	5.32	7.39
	Attendant	11	8.55	2.296	7.00	10.09
	Artisan	10	8.00	2.749	6.03	9.97
	Fitter	7	7.86	3.579	4.55	11.17
	Operator	9	7.33	3.041	5.00	9.67
	Engineer	11	5.09	2.914	3.13	7.05

Noise level LAeq	Shift Supervisor	7	89.171	11.7237	78.329	100.014
	Technician	10	92.150	11.4088	83.989	100.311
	Attendant	3	95.200	3.0414	87.645	102.755
	Artisan	6	98.183	2.3310	95.737	100.630
	Fitter	1	96.900			
	Operator	6	91.417	11.5574	79.288	103.545
	Engineer	4	78.475	14.9734	54.649	102.301
	Shift Supervisor	7	141.857	3.2134	138.885	144.829

Peak level	Technician	10	137.470	12.3516	128.634	146.306
	Attendant	3	138.100	3.4641	129.495	146.705
	Artisan	6	143.617	3.7280	139.704	147.529
	Fitter	1	145.500			
	Operator	6	141.833	3.6401	138.013	145.653
	Engineer	4	130.725	18.1744	101.805	159.645

### Previous Illnesses Affecting Hearing Loss

Seven (6.6) participants in the gas-fired electric plant were hypertensive. One (0.9) participant had had severe ear infection/injury at childhood. Two (1.9) participants had diabetes. Nine (8.6) participants had experienced ear pain prior to time of the study. One (0.9) participant had used an ototoxic drug, in this case quinine, two weeks before the study (Table 4).

**Table 4:** Previous illnesses affecting hearing loss reported by participants.

		Plant A n = 32	Plant B n = 33	Plant C n = 41	All n = 106

Severe ear infection/injury at childhood	Yes	1(3.1)	0(0.0)	0(0.0)	1(0.9)
	No	31(96.9)	33(100.0)	41(100.0)	105(99.1)

High blood pressure	Yes	1(3.1)	2(6.1)	4(9.8)	7(6.6)
	No	31(96.9)	31(93.9)	37(90.2)	99(93.4)

Diabetes	Yes	0(0.0)	1(3.0)	1(2.4)	2(1.9)
	No	32(100.0)	32(97.0)	40(97.6)	104(98.1)

Ear pain	Yes	2(6.3)	2(6.1)	5(12.5)	9(8.6)
	No	30(93.8)	31(93.9)	35(87.5)	96(91.4)

Use of ototoxic drugs	Yes	1(3.1)	0(0.0)	0(0.0)	1(0.9)
	No	31(96.9)	33(100.0)	41(100.0)	105(99.1)

### Self-Reported Noise-induced Hearing Loss

Fifty-seven (53.8) reported feeling hearing loss, 44(41) reported to have difficulty in hearing people speaking during conversation, 36(34.0) need to shout in order to be understood when standing three feet away from them, 55(51.9) participants watch people’s faces when they speak to understand what is being said, furthermore 48(45.3) participants reported responding inappropriately in a conversation (Table 5).

**Table 5:** Self-reported noise-induced hearing loss.

Self-reported noise-induced hearing loss (NIHL)		**Plants n(%)**			

		Plant A n = 32	Plant B n = 33	Plant C n = 41	All n = 106

Feeling hearing loss	Never	18(56.2)	20(60.6)	11(26.4)	49(46.2)
	Yes	14(43.8)	13(39.4)	30(73.2)	57(53.8)
Difficulty hearing people speaking during conversation	Never	24(75.0)	24(72.7)	14(34.1)	62(58.5)
	Yes	8(25.0)	9(27.3)	27(65.9)	44(41.5)

Shouting in order to be understood when you are at three feet away.	Never	23(71.9)	25(75.8)	22(53.7)	70(66.0)
	Yes	9(28.1)	8(24.2)	19(46.3)	36(34.0)

Watching people’s face when they speak	Never	17(53.1)	17(51.5)	17(41.5)	51(48.1)
	Yes	15(46.9)	16(48.5)	24(58.5)	55(51.9)

Respond inappropriately in a conversation	Never	17(53.1)	23(69.7)	18(43.9)	58(54.7)
	Yes	15(46.9)	10(30.3)	23(56.1)	48(45.3)

Logistic regression analysis shows that there is association between age and feeling of hearing loss where p value is 0.005 and odds ratio OR 1.056; 95% C.I (1.017–1.097) among gas-fired electric plant workers. Daily duration of exposure to noise was associated with difficulty in hearing during conversation where p value is 0. 045 (data not shown).

Workers in plant C were more likely to report difficulties in hearing conversations compared to workers from plant B and A (AOR 4.22; 1.43–12.42) adjusted for age, ear pain and habit of attending disco. Furthermore, workers at plant C had higher crude odds ratio (3.51; 1.319.37) of reporting feeling of hearing loss (Table 6).

**Table 6:** Predictors and self-reported noise-induced hearing loss.

Symptoms		Crude odds ratio (95% C.I)	Adjusted odds ratio (95% C.I)

Feeling hearing loss	Plant A	ref	–
	Plant B	0.84 (0.31–2.24)	0.49 (0.16–1.51)
	Plant C	3.51 (1.31–9.37)	2.47 (0.82–7.39)

Difficulty in hearing during conversation	Plant A	Ref	–
	Plant B	1.13 (0.37–3.41)	0.83 (0.26–2.68)
	Plant C	5.79 (2.07–16.18)	4.22 (1.43–12.42)

People need to shout in order to be understood when three feet away from you	Plant A	Ref	–
	Plant B	0.82 (0.27–2.48)	0.56 (0.18–1.89)
	Plant C	2.21 (0.82–5.91)	1.46 (0.50–4.23)

Watch people when speaking at three feet away	Plant A	Ref	–
	Plant B	1.07 (0.40–2.82)	1.03 (0.37–2.90)
	Plant C	1.60 (0.63–4.06)	1.29 (0.4–3.57)

Odds ratio adjusted for ear pain, age and habit of attending disco.

### Other Symptom of Noise-induced Hearing Loss

About 21(19.8) n = 106 participants reported ringing or buzzing sounds in their ears. Higher prevalence of tinnitus was observed among workers in plant C compared to the other plants (p = 0.048) (Table 7).

**Table 7:** Symptom of NIHL (Tinnitus).

		Plant Address (n (%))			All	p- value*	X^2^ Value
			
		Plant A	Plant B	Plant C			

Ringing or buzzing sounds in your ears	Yes	5(15.6)	3(9.1)	13(31.7)	21(19.8)		
	No	27(84.4)	30(90.9)	28(68.3)	85(80.2)	0.048	6.030

^*^Fischer’s exact

### Hearing Protective Devices (HPDS)

A majority of participants in gas-fired electric plants – 101(95.3) – use hearing protective devices. About 8(7.5) participants use ear plugs, 41(38.7) participants use ear muffs and 57(53.8) participants use both ear plug and ear muff. About 85(80.2) participants had training on the hearing protective devices. Sixty-six (62.3) participants were required by their managers to use hearing protective devices in the gas-fired electric plant. About 84(79.2) participants always use hearing protective devices when working in gas-fired electric plants (Table 8).

**Table 8:** Proportion of HPDs users in gas-fired electric plants.

Plants n(%)					

USE of HPDs		Plant A n = 32	Plant B n = 33	Plant C n = 41	All n = 106

Use of HPDs	Yes	28(87.5)	32(97.0)	41(100.0)	101(95.3)
	No	4(12.5)	1(3.0)	0(0.0)	5(4.7)

Type of Hearing protective device	Ear Plug	0(0.0)	0(0.0)	8(19.5)	8(7.5)
	Ear Muff	29(90.6)	7(21.2)	5(12.2)	41(38.7)
	Both	3(9.4)	26(78.8)	28(68.3)	57(53.8)

Training	Yes	28(87.5)	29(87.9)	28(68.3)	85(80.2)
	No	4(12.5)	4(12.1)	13(31.7)	21(19.8)

Enforcement on the use of HPDs	Yes	18(56.3)	22(66.7)	26(63.4)	66(62.3)
	No	14(43.8)	11(33.3)	15(36.6)	40(37.7)

Duration of use of HPDs	Sometimes	17(53.1)	3(9.1)	2(4.9)	22(20.8)
	Always	15(46.9)	30(90.9)	39(95.1)	84(79.2)

## Discussion

Personal noise exposure level among gas-fired electric plant workers were 89.9dB(A), 101.8dB(A) and102.6dB(A) in plants A, B and C respectively, above OEL of 85dB(A) set by Tanzanian Bureau of Standards [22] and the World Health Organization [23]. A feeling of hearing loss was reported by 53.8% of the participants. Workers from factory C had higher odds of reporting difficulties hearing during conversations. Age and daily duration of exposure had significant association with reported noise-induced hearing loss. There are no studies done in electrical plants in Tanzania; however, similar exposure to high noise level has been reported by the study done by Mbuligwe in wood and metal works industries in Dar es Salaam [4]. The maximum noise level measured at the gas-fired electric plants A, B and C were 96.7, 102.2 and104.2dB(A), respectively. The values from this study are higher than those reported in a study done by Sean on occupational noise exposure assessment for coal and natural gas power plant workers where the highest value, 93.8dB(A) after 8 hours and 13 minutes of run time, came from one of the employees working at the coal power plant [24].

Using a short screening validated noise exposure questionnaire, the mean score for reported noise exposure for all the gas-fired electric plants was AM(SD) was 6.9(3.3). Gas plant C had a higher mean score of 8.59(2.8), whereas gas plant B had a mean noise score of 7.1(2.7) and gas plant A had the mean noise score of 4.5(3.1). A majority of the participants, 74(69.8), had a higher score, showing possible high exposure to noise. Plant A had a few (37.5%) who had a high noise score compared to plants B and C (79%) and (88%), respectively.

There was a significant correlation between noise score and noise exposure level among gas-fired electric plant workers (r = 0.409 and p = 0.012). This shows that for developing countries like Tanzania, where there is shortage of personal noise exposure dosimeter, a one-minute validated questionnaire can be used to report noise exposure in occupational settings.

Participants in both operation and maintenance departments had a higher equivalent noise exposure level (LAeq) measured over eight hours of 101.980 ± 3.6dB(A) followed by maintenance department had equivalent noise exposure level (LAeq) measured over eight hours of 98.5 ± 12.4dB(A) and operation department had equivalent noise exposure level (LAeq) measured over eight hours of 97.7 ± 8.8dB(A). This has been presented in another study [24], where maintenance employees were subjected to the greatest TWA workplace noise exposure regardless of plant type, probably because of the increased duration of noise exposure that workers receive during the performance of maintenance activities in the proximity of loud equipment.

Among the study participants in gas-fired electric plants, 57(53.8) reported feeling hearing loss. This prevalence is higher than that reported by a study conducted in Dar es Salaam and Morogoro, Tanzania [25], whereby hearing problems reported to be 6.8% (95% CI 4.01–10.6%, n = 250). The differences between the studies could be due to methodologies such as different sets of question used. Globally, 16% of the disabling hearing loss in adults is attributed to occupational noise, the statistics ranging from 7% to 21% [13]. However, the studies conducted in Africa reported hearing loss proportions ranging from 23.4% up to a shocking 79.8%. Self-reported hearing loss has been documented at 23.76% (24) among market mill workers, while hearing loss, as determined audiometrically, was found in 43.56% (44) market mill workers [26]. This indicates that if hearing loss will be determined audiometricaly in a similar study sample, the prevalence of hearing loss might be higher compared to self-reported hearing loss.

About 21(19.8) participants reported ringing or buzzing in their ears in the current study, which is low compared to the study done among construction workers in the United States of America, where about 38% indicated they had ringing or buzzing in their ears [27]. This is probably due to long-term exposure to noise that has damaged hearing.

Forty-eight (45.3) participants reported a problem with responding inappropriately during conversation due to a problem of not understanding what people say during conversation in noisy environments. This is low compared to a proportion of 60% who reported a problem understanding what people say in noisy environments in a study done among construction workers in the United States of America [27]. About 44(41.5) participants reported difficulty hearing people during conversations; this proportion is low compared to a study done among construction workers in the United States of America, where a large proportion (62%) of OEs in this study reported difficulty in understanding people’s conversation in noisy environment [27]. A majority of the participants in the gas-fired electric plants – 101(95.3) – use hearing protective devices. The high percentage of use of hearing protective devices among gas-fired electric plant workers complies with the TBS and ILO guidelines, which require that at the noise level above 85dB(A) and exposure time of eight hours daily, all employees should be provided with hearing protectors [22]. About 54(53.5) participants who reported using hearing protective devices also reported feeling hearing loss; this indicates that perhaps they were using the wrong protective devices with less reduction capacity while the mean peak noise exposure level was 139.6dB (A). In this study we did not test the capacity of the HPDs. Findings reported in this study are different from previous studies, where by a study done among construction workers showed protective effect of using HPDs; workers who reported frequent use of HPDs had significantly better hearing [27]. In the study with Korean airport workers exposed to high noise (>85dB(A), 8-h TWA), a study done by Hong et al. found that workers who used HPDs consistently had significantly less hearing loss than those who did not [28]. A more recent study in Canada reported that construction workers who always wore HPDs showed better hearing, compared to those who did not [29]. In our study, HPDs – ear muffs and plugs – were being worn interchangeably regardless of the level of the noise. This could also be the reason for less protection at noises above 115dB(A).

## Conclusion

Workers in gas-fired electrical plants are exposed to significant high noise levels, above 85dB(A), which could impair their hearing capacity. Artisans and technicians received significantly higher amounts of exposure than other job categories. The measured noise levels (TWA and peak level) were found to be higher than the TBS and WHO acceptable limit in some production sections. This suggests that specific intervention is required to protect workers exposure to noise and health effects at the work place.

## Author informations

Witness John participated in the conception of the study, proposal development, data collection analysis and writing of the manuscript. Drs Gloria Sakwari and Simon Mamuya participated in proposal development, data analysis and writing of the manuscript.
